# Early resolution of subretinal fluid without high-dose corticosteroids in a pregnant patient with Vogt-Koyanagi-Harada disease: a case report

**DOI:** 10.1186/s12348-015-0050-3

**Published:** 2015-06-27

**Authors:** Keijiro Sugita, Kyoichi Mizumoto, Nahoko Kato, Masahiro Zako

**Affiliations:** Department of Ophthalmology, Aichi Medical University, Nagakute, Aichi 480-1195 Japan

**Keywords:** High-dose corticosteroids, Pregnancy, Vogt-Koyanagi-Harada disease

## Abstract

**Background:**

At present, there is no standard of treatment using systemic high-dose corticosteroids in cases of pregnant women with Vogt-Koyanagi-Harada (VKH) disease. Although high-dose systemic corticosteroid treatment is often used for VKH disease during pregnancy, it also poses a risk to the fetus.

**Findings:**

A 29-year-old woman in the 34th week of pregnancy experienced bilateral metamorphopsia. She had been receiving 5 mg of prednisolone daily for the past 8 years as treatment for rheumatoid arthritis. In order to prevent progression of bilateral serous retinal detachment caused by VKH disease, we recommended the use of high-dose systemic corticosteroids but the patient refused. Thus, we administered only topical ophthalmic betamethasone for mild anterior uveitis. Surprisingly, however, the bilateral bullous retinal detachment healed in just 19 days after the onset of symptoms. A healthy baby was born 1 month later, and sunset glow fundus was subsequently observed without any recurrence of uveitis.

**Conclusions:**

We report a case in which bilateral subretinal fluid caused by VKH disease in a young woman during late pregnancy resolved without high-dose corticosteroid treatment. Pregnancy may have had a beneficial effect on uveitis activity caused by VKH disease. To our knowledge, this report describes the shortest healing period for bilateral bullous retinal detachment in a pregnant woman with VKH disease.

## Findings

### Introduction

Although reports have described cases of pregnant women with Vogt-Koyanagi-Harada (VKH) disease [1–10], no standard of treatment that includes systemic high-dose corticosteroids has been established. Previously, a 24-year-old pregnant woman with VKH disease successfully healed on the 26th day after onset without the use of systemic or topical corticosteroids [1]. Here, we report a 29-year-old pregnant woman who received prednisolone 5 mg/day for rheumatoid arthritis; she exhibited bullous retinal detachment caused by VKH disease, and the subretinal fluid successfully resolved in just 19 days after the onset of symptoms. To our knowledge, this report describes the shortest healing period for bilateral bullous retinal detachment in a pregnant woman with VKH disease.

### Case report

**Fig. 1:**
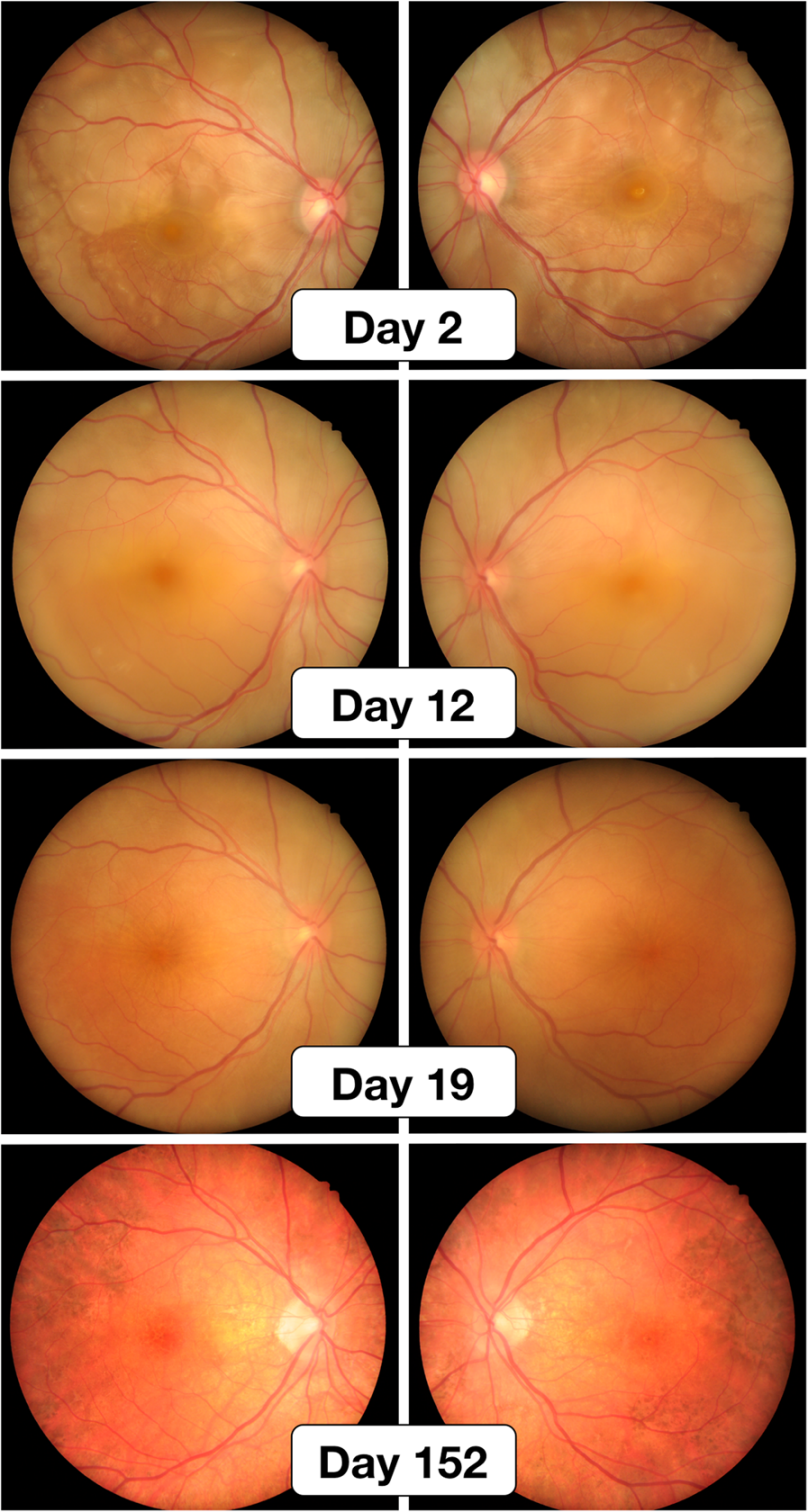
Color fundus photographs, taken starting the day after the patient’s initial symptoms (bilateral acute vision loss with metamorphopsia) appeared. Multi-focal serous retinal detachment was apparent on day 2 and then reached bullous retinal detachment on day 12. Without high-dose systemic corticosteroid treatment, subretinal fluid had nearly disappeared by day 19, and sunset glow fundus was observed on day 152 without any recurrence of uveitis

**Fig. 2:**
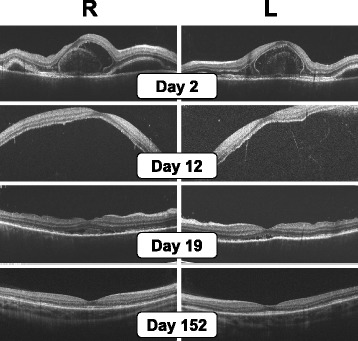
Spectral domain optical coherence tomography on days 2, 12, 19, and 152. Progression and resolution of bilateral serous retinal detachment caused by VKH disease. *R* right eye, *L* left eye

On 1 November 2013, a 29-year-old Japanese woman in the 34th week of her first pregnancy began to experience acute vision loss in both eyes, coupled with the appearance of metamorphopsia. She visited our hospital on the following day with bilateral blurred vision with meningismus. The patient’s best corrected visual acuity (BCVA) was 0.5 in her right eye and 0.3 in her left eye. The patient had mild iridocyclitis in both eyes. Ophthalmoscopic examination revealed multi-focal serous retinal detachment (Figs. 1 and 2, day 2). The intraocular pressures were 18 mmHg (right eye) and 12 mmHg (left eye). B-scan ultrasonography was not performed, and choroidal thickness was unclear. The patient refused fluorescein angiography. Her blood pressure was 116/60. Based on these clinical findings and the revised criteria for diagnosis of VKH [11], she was diagnosed with incomplete VKH. We recommended the use of systemic high-dose corticosteroids for the treatment of VKH disease. However, the patient refused treatment because she was worried about adverse effects to the fetus. We therefore administered only topical ophthalmic betamethasone. Because she had rheumatoid arthritis, the patient had been receiving prednisolone 5 mg daily for the past 8 years. The patient had no history of any other medical disorders, including hypertension. She was a non-smoker and did not drink alcohol while pregnant. Her pregnancy had, up to this point, been uneventful. She underwent all routine laboratory and ultrasound tests, and the fetus had developed normally. Blood cortisol levels during pregnancy were not investigated.

On 12 November 2013, the patient returned, presenting with progressive vision loss in both eyes that had begun 4 days earlier. The patient’s BCVA was 0.3 in her right eye and 0.2 in her left eye. Ophthalmic examination revealed bullous retinal detachment in both eyes (Figs. 1 and 2, day 12). Intraocular pressures were 10 mmHg (right eye) and 11 mmHg (left eye). However, she continued to refuse treatment. At a subsequent visit on 19 November 2013, she reported visual improvement in both eyes. Ophthalmic examination on that day revealed the disappearance of subretinal fluid from both eyes (Figs. 1 and 2, day 19). Her BCVA was 0.8 in her right eye and 0.9 in her left eye. Intraocular pressures were 19 mmHg (right eye) and 16 mmHg (left eye). On 27 November 2013, she delivered a healthy female baby (gestational age 37 weeks and 5 days, 2294 g) by spontaneous vaginal delivery. The baby’s birth weight was low, but not small for her gestational age. A subsequent examination on 1 April 2014 revealed that the patient’s BCVA was 1.0 in both eyes, and examination of her fundus revealed typical sunset glow fundus, without subretinal fluid (Figs. 1 and 2, day 152). Intraocular pressures were 14 mmHg (right eye) and 10 mmHg (left eye). She exhibited alopecia and poliosis. After reviewing all of the patient’s clinical findings, we diagnosed her with complete VKH. On 11 May 2015, our final meeting with the patient, she did not exhibit any recurrence of VKH. The patient was still receiving 5 mg prednisolone daily for rheumatoid arthritis. Prior to and during her pregnancy, the patient’s blood pressure stayed within normal limits, and she never exhibited hypertension.

### Discussion

High-dose systemic corticosteroid treatment is often used for VKH disease during pregnancy [2–7, 10], although some reports indicate that it may also pose a risk to the fetus. In one such report, Ohta et al. described a case of fetal death in a 28-year-old Japanese woman who was being treated with intravenous high-dose prednisolone for VKH disease [10]. In another report, Steahly detailed the adverse effects of high-dose corticosteroid treatment for VKH disease during pregnancy in two cases: one patient suffered a spontaneous abortion, and the other delivered a premature infant [5]. Doi et al. also reported that a pregnant VKH patient treated with a high-dose systemic corticosteroid delivered a low-birth-weight infant with an epibulbar dermoid, lipodermoids, and preauricular appendages, although the relationship among VKH disease, systemic corticosteroids, spontaneous abortion, and congenital malformation was not clear [6]. Østensen and Skomsvoll stated that, administered during early pregnancy, corticosteroids are also considered a risk factor for the development of oral clefts [12]. In light of these potential complications, they have proposed that the daily dose should be kept to ≤15 mg during the first trimester. Miyata et al. suggested that treatment should be chosen according to the severity of inflammation, the stage of pregnancy, maternal condition, and fetal condition. In their report, successful treatment of three cases of VKH disease during pregnancy was demonstrated [7]. One patient with mild inflammation was treated with a topical corticosteroid, while two additional cases were treated with a high-dose systemic corticosteroid. No abnormalities were observed during or after the deliveries.

The use of high-dose corticosteroids during pregnancy is associated with premature rupture of the membranes, intrauterine growth restriction, and the precipitation of maternal complications such as gestational diabetes, hypertension, osteoporosis, and avascular bone necrosis [13]. It should be also noted that prednisone, prednisolone, and methylprednisolone have minimal placental transfer and are the drugs of choice during pregnancy. On the other hand, fluorinated corticosteroids such as dexamethasone and betamethasone easily cross the placenta and therefore should not be used unless there is intent to treat the fetus [13].

Although an early pregnancy flare-up is typical of VKH disease [8], pregnancy may have a beneficial effect on uveitis activity caused by VKH disease [9]. Generally, during early pregnancy, cellular immunity is suppressed and the production of inflammatory cytokines is low, while corticosteroid production is high. Because of these immunological conditions, the inflammation caused by VKH disease during the first and second trimesters of pregnancy may be mild. Consequently, in our case, VKH disease may not have appeared during the first and second trimesters. In addition, the period of time required for bilateral absorption of the subretinal fluid was obviously shorter than in normal VKH patients, even without high-dose corticosteroid treatment. Steahly showed that some cases of VKH disease improved clinically during pregnancy after the discontinuation of corticosteroid treatment; however, symptoms recurred after their pregnancies ended [5]. These findings suggest it might be unnecessary to administer more corticosteroid to treat persistent serous retinal detachment after systemic corticosteroid treatment.

The patient had received 5 mg prednisolone daily for the past 8 years as treatment for rheumatoid arthritis. It is unclear whether this daily oral administration of 5 mg prednisolone contributed to the resolution of VKH in this patient; however, we do not believe 5 mg of oral prednisolone is enough to resolve the active phase of VKH as shown in this case. The precise mechanism is not clear, as we did not investigate this patient’s serum hormone levels during pregnancy and neither did her gynecologist or rheumatologist.

In summary, we report a 29-year-old woman in the 34th week of pregnancy taking prednisolone 5 mg per day, who exhibited bilateral bullous retinal detachment as a result of VKH disease. Without administration of high-dose systemic corticosteroids, subretinal fluid resolved 19 days after the onset of symptoms, and uveitis did not recur thereafter. No standard of treatment has been established for pregnant patients with VKH disease. In the absence of systemic high-dose corticosteroid treatment, pregnancy may favorably influence and modulate the course of the disease, as exemplified by this patient.

### Consent

We obtained written informed consent from the patient to publish this report and any accompanying images. Our own institute’s IRB also granted approval.

## Abbreviations

BCVA: best corrected visual acuity
VKH: Vogt-Koyanagi-Harada

## References

[1] Nohara M, Norose K, Segawa K (1995). Vogt-Koyanagi-Harada disease during pregnancy. Br J Ophthalmol.

[2] Friedman Z, Granat M, Neumann E (1980). The syndrome of Vogt-Koyanagi-Harada and pregnancy. Metab Pediatr Ophthalmol.

[3] Matsubara S, Kuwata T, Ohkawara Y, Makino S (2011). Headache in late pregnancy: a symptom for Vogt-Koyanagi-Harada disease. Arch Gynecol Obstet.

[4] Tien MC, Teoh SC (2009). Treatment of Vogt-Koyanagi-Harada syndrome in pregnancy. Can J Ophthalmol.

[5] Steahly LP (1990). Vogt-Koyanagi-Harada syndrome and pregnancy. Ann Ophthalmol.

[6] Doi M, Matsubara H, Uji Y (2000). Vogt-Koyanagi-Harada syndrome in a pregnant patient treated with high-dose systemic corticosteroids. Acta Ophthalmol Scand.

[7] Miyata N, Sugita M, Nakamura S, Isobe K, Matoba H, Tsuda K, Tanaka K, Ohno S (2001). Treatment of Vogt-Koyanagi- Harada’s disease during pregnancy. Jpn J Ophthalmol.

[8] Rabiah PK, Vitale AT (2003). Noninfectious uveitis and pregnancy. Am J Ophthalmol.

[9] Snyder DA, Tessler HH (1980). Vogt-Koyanagi-Harada syndrome. Am J Ophthalmol.

[10] Ohta K, Gotoh N, Yonezawa H, Oka K, Ashida T (2007). Fetal death following high-dose systemic steroid therapy in a pregnant patient with Vogt-Koyanagi-Harada disease. Nihon Ganka Gakkai Zasshi.

[11] Rao NA, Sukavatcharin S, Tsai JH (2007). Vogt-Koyanagi-Harada disease diagnostic criteria. Int Ophthalmol.

[12] Østensen ME, Skomsvoll JF (2004). Anti-inflammatory pharmacotherapy during pregnancy. Expert Opin Pharmacother.

[13] Mok CC, Wong RW (2001). Pregnancy in systemic lupus erythematosus. Postgrad Med J.

